# Comparison of pregnancy outcome after adding oral or intramuscular progesterone to vaginal progesterone in frozen embryo transfer: A cross-sectional study

**DOI:** 10.18502/ijrm.v22i10.17661

**Published:** 2024-12-02

**Authors:** Sahereh Arabian, Maryam Eftekhar, Saeideh Dashti, Nahid Homayoon, Elham Nikfarjam

**Affiliations:** ^1^Research and Clinical Center for Infertility, Yazd Reproductive Sciences Institute, Shahid Sadoughi University of Medical Sciences, Yazd, Iran.; ^2^Gynecology and Infertility Department, Shiraz Fertility Center, Shiraz, Iran.

**Keywords:** Assisted reproductive technology, Luteal phase, Progesterone, Embryo transfer.

## Abstract

**Background:**

Currently, frozen embryo transfers (FET) account for 41% of all embryo transfer cycles. Vaginal progesterone preparations have become the leading choice for luteal phase support due to their convenient application; however, using only vaginal progesterone during FET cycles results in a lower ongoing pregnancy rate.

**Objective:**

This study aimed to investigate whether replacing intramuscular (IM) progesterone with oral dydrogesterone in FET cycles affects pregnancy outcomes or not.

**Materials and Methods:**

In this cross-sectional study, pregnancy outcomes were analyzed in women who underwent cleavage stage FET during an endometrial preparation cycle using hormone replacement therapy at Yazd Reproductive Sciences Institute, Yazd, Iran, between April 2023 and November 2023. The study examined 2 groups based on a luteal phase support regimen: the dydrogesterone group, which received vaginal progesterone and oral dydrogesterone, and the IM progesterone group, which received vaginal progesterone and IM progesterone. Data were extracted from patient files to compare outcomes between the 2 groups.

**Results:**

A total of 960 cycles meeting the inclusion criteria were analyzed, with 292 women in the dydrogesterone group and 668 women in the IM progesterone group, and pregnancy outcomes were compared between the 2 groups. The chemical pregnancy rates (28.4% vs. 29.9%, p = 0.636), clinical pregnancy rates (25.3% vs. 26.9%, p = 0.604), and ongoing pregnancy rates (21.9% vs. 23.8%, p = 0.525) were lower and miscarriage rates (14.7% vs. 11.7%, p = 0.210) were higher in dydrogesterone group compared to IM progesterone group, although this difference was not statistically significant.

**Conclusion:**

Based on the ease of use and similar pregnancy outcomes of oral dydrogesterone, it can potentially replace the daily injections of IM progesterone.

## 1. Introduction

Currently, frozen embryo transfers (FET) account for 41% of all embryo transfer cycles (1). During FET, a fertilized embryo can be transferred into the uterus, either, naturally during a spontaneous ovulation cycle or in an artificial cycle. In artificial cycles, ovulation is intentionally suppressed using exogenous steroids to precisely control the hormonal environment for implantation. Unlike natural cycles, artificial cycles involve no follicular or corpus luteum development. Instead, exogenous steroids are administered to create an optimal endometrium and support early pregnancy (2, 3).

Several estrogen and progesterone preparations can be used in FET cycles. Once the endometrium reaches a suitable thickness, progesterone is given to prepare it for implantation and early pregnancy support. Progesterone may be administered by oral, intramuscular (IM), intravaginal, subcutaneous, or rectal routes. Vaginal progesterone preparations have risen to become the leading choice for luteal phase support (LPS) due to their convenient application. IM progesterone requires daily injections, which can be painful (4). Despite oral administration's convenience, oral micronized progesterone has limitations for LPS in in-vitro fertilization (IVF) due to its low bioavailability. The medication undergoes extensive first-pass metabolism, significantly reducing its effectiveness (5–7).

Dydrogesterone, a stereoisomer of progesterone, offers a significant advantage for LPS in IVF: its high bioavailability. Dydrogesterone's unique chemical structure, a “curved isomer", allows it to bypass the extensive first-pass metabolism that significantly reduces the effectiveness of orally administered micronized progesterone (8).

Some studies have shown that adding IM progesterone to vaginal progesterone increases the chance of pregnancy (9–13). However, IM injections are not patient-friendly and may be associated with complications such as abscess formation at the site of injection (4). The purpose of our study was to find whether replacing IM progesterone with oral progesterone in women receiving vaginal progesterone for LPS is associated with similar pregnancy outcomes.

## 2. Materials and Methods

### Study design and population

In this cross-sectional study, data were extracted from the electronic medical records of all women who underwent FET using cleavage-stage embryos at Yazd Reproductive Sciences Institute, Yazd, Iran between April 2023 and November 2023. The inclusion criteria were limited to cases involving cleavage-stage embryo transfer during an endometrial preparation cycle using hormone replacement therapy. Exclusion criteria included medical records with missing data and cases in which endometrial preparation was conducted using a natural ovulatory cycle or ovulation induction cycle.

Among the 1042 FET cycles reviewed, 82 were excluded due to embryo degeneration after thawing, missing data, or endometrial preparation using a natural cycle. The remaining 960 cycles were analyzed, with 292 cases in the dydrogesterone group and 668 cases in the IM progesterone group.

Data extracted from participants' medical records included baseline and cycle characteristics including age, body mass index (BMI), duration and type of infertility, serum anti-Mullerian hormone levels, number of previous implantation failures, endometrial thickness on the day progesterone was started, duration of the FET cycle, number of embryos transferred, and total number of good-quality embryos transferred. Additionally, pregnancy outcomes, including chemical, clinical, and ongoing pregnancies, as well as miscarriage rates, were also recorded.

A chemical pregnancy was diagnosed to check if the level of serum beta-human chorionic gonadotropin was at least 50 IU/L, 14 days after the embryo transfer. Clinical pregnancy was confirmed by detecting fetal heart activity on ultrasound 4 wk after the embryo transfer. A pregnancy was considered ongoing if it had been established after the 12 ${\;}^{\text{th}}$ wk of gestation. A gestational sac or fetal heartbeat loss in clinically pregnant women before the 13 ${\;}^{\text{th}}$ wk was defined as an early abortion (14).

### Endometrial preparation protocol

In preparation for FET, after confirmation of the absence of ovarian cyst by performing vaginal ultrasound on the first or second day of menstruation, all participants commenced oral estradiol valerate 6 mg per day, beginning on day 2 of the cycle. On day 13, a transvaginal ultrasound was performed to assess endometrial thickness. Progesterone administration was begun if it was ≥ 7 mm (9).

Women were administered either vaginal progesterone 400 mg twice daily along with oral dydrogesterone 10 mg twice daily (dydrogesterone group), or vaginal progesterone 400 mg twice daily combined with IM progesterone 50 mg daily (IM progesterone group), depending on physician's decision or women's preference. There were 292 cases in the dydrogesterone group and 668 cases in the IM progesterone group. The embryos were transferred 3 days after progesterone was initiated. Up to 2 or 3 cleavage-stage embryo was transferred.

### Ethical Considerations

This study was approved by the Ethics Committee of Yazd Research and Clinical Center for Infertility, Shahid Sadoughi University of Medical Sciences, Yazd, Iran (Code: IR.SSU.RSI.REC.1402.105). The study was conducted retrospectively, and the data of participants was kept confidential to use only for research purposes.

### Statistical Analysis

The statistical package for the social science version 26 for Windows (SPSS Inc., Chicago, IL, USA) was applied for data analysis. For continuous variables, the Student's *t* test was used to compare differences between groups assuming a normal distribution. Alternatively, the Mann-Whitney U test was used if normality was not assumed. Categorical variables were analyzed using the Chi-square test for larger sample sizes or the Fisher's exact test for smaller sample sizes or sparse data. Data were presented as mean ± SD for continuous variables and number (%) for categorical variables. A significance level of p < 0.05 was used in this study.

## 3. Results

FET cycles were reviewed at the Research and Clinical Center for Infertility, Yazd Reproductive Sciences Institute, Yazd, Iran. Out of 1042 cycles investigated, 82 were excluded due to embryo degeneration after thawing, missing data, and endometrial preparation using a natural ovulatory cycle and ovulation induction cycle.

The remaining 960 cycles were analyzed as 292 cases in the dydrogesterone group and 668 cases in the IM progesterone group.

No statistically significant difference was found between baseline characteristics including age, BMI, anti-Mullerian hormone level, duration and type of infertility, and number of prior FET (Table I). No statistically significant differences were observed between the 2 groups in endometrial thickness, cycle duration, number of embryos transferred, or the total number of good-quality embryos transferred (Table II). The rates of chemical pregnancy, clinical pregnancy, ongoing pregnancy, and early miscarriage for both groups are presented in table III. Notably, no statistically significant differences were observed between the groups for any of these assisted reproductive technology (ART) outcomes.

**Table 1:** Baseline characteristics of study groups

**Variables**		**Dydrogesterone (n = 292)**	**IM progesterone (n = 668)**	**P-value**
Age (yr)*		33.86 ± 5.99	33.56 ± 6.03	0.482
BMI (kg/m^2^)*		25.86 ± 4.46	26.16 ± 4.99	0.384
Duration of infertility (yr)**		7.99 ± 5.10 (7.00, 7.00)	7.76 ± 4.67 (7.00, 7.00)	0.832
Type of infertility***				
	**Primary**	213 (72.9)	491 (73.5)	
	**Secondary**	79 (27.1)	177 (26.5)	0.857
AMH**		3.67 ± 3.51 (2.70, 4.03)	4.16 ± 3.87 (3.05, 4.70)		0.090
No. of implantation failure***				
	**0**	104 (35.6)	219 (32.8)	
	**1**	98 (33.6)	219 (32.8)	
	**2**	48 (16.4)	132 (19.7)	
	**≥ 3**	42 (14.4)	98 (14.7)	0.631
*Data presented as Mean ± SD, Independent samples *t* test. **Data presented as Mean ± SD (Median, interquartile range), Mann-Whitney test. ***Data presented as n (%), Chi-square tests. IM: Intramuscular, BMI: Body mass index, AMH: Anti-Mullerian hormone				

**Table 2:** Cycle characteristics of study groups

**Variables**	**Dydrogesterone (n = 292)**	**IM progesterone (n = 668)**	**P-value**
Endometrial thickness (mm)*	8.88 ± 1.48 (8.60, 1.20)	8.92 ± 1.44 (8.80, 1.60)	0.137
Cycle duration, days*	16.73 ± 1.50 (17.00, 2.00)	16.90 ± 1.53 (17.00, 2.00)	0.442
No of transferred embryos*	1.92 ± 0.35 (2.00, 0.00)	1.90 ± 0.36 (2.00, 0.00)	0.489
No. of transferred good quality embryos**	262 (89.7)	581 (87.1)	0.231
*Data presented as Mean ± SD (median, interquartile range), Mann-Whitney test. **Data presented as n (%), Chi-square tests. IM: Intramuscular			

**Table 3:** ART outcomes in study groups

**Variables**	**Dydrogesterone (n = 292)**	**IM progesterone (n = 668)**	**P-value**
**Chemical pregnancy**	83 (28.4)	200 (29.9)	0.636
**Clinical pregnancy**	75 (25.3)	180 (26.9)	0.604
**Ongoing pregnancy**	64 (21.9)	159 (23.8)	0.525
**Miscarriage rate**	11/75 (14.7)	21/180 (11.7)	0.510
Data presented as n (%), Chi-square test. ART: Assisted reproductive technology, IM: Intramuscular			

## 4. Discussion

The present study was designed to determine whether replacing IM progesterone with oral dydrogesterone is associated with similar pregnancy outcomes in FET cycles. Oral dydrogesterone was found to be as effective as IM progesterone in maintaining pregnancy, as measured by chemical pregnancy rate, clinical pregnancy rate, ongoing pregnancy rate, and miscarriage rate.

Even though there are no established guidelines, vaginal progesterone is the most commonly used method of LPS (4, 15). A randomized controlled trial compared IM progesterone, vaginal progesterone, and a combination of both. The study found that using only vaginal progesterone during FET cycles resulted in lower ongoing pregnancy rate (12). The results of this study were similar to the results of a study conducted in our center and showed that combining vaginal progesterone with IM progesterone may lead to better pregnancy outcomes compared to using vaginal progesterone alone (9). At our clinic, we combine vaginal progesterone with either IM progesterone or oral dydrogesterone. Oral dydrogesterone offers much better tolerability compared to IM injections, obviously due to pain and inflammation at the injection site (4). Our findings support the growing body of research demonstrating the effectiveness of dydrogesterone for LPS in ART cycles (16–19). Of note, the studies mentioned used varying daily doses of dydrogesterone, ranging from 20–30 mg. Additionally, these studies compared dydrogesterone with different progesterone preparations, making direct comparisons difficult. In a randomized controlled trial 162 IVF candidates were evaluated for pregnancy rates, adverse reactions, and medication costs associated with oral dydrogesterone vs. micronized vaginal progesterone (MVP). The study demonstrated that dydrogesterone had statistically similar chemical pregnancy rates, clinical pregnancy rates, ongoing pregnancy rates, miscarriage rates, and safety profiles compared to MVP (17). A study comparing 5 different hormone treatments for LPS in women undergoing FET found that adding dydrogesterone to micronized progesterone gel resulted in higher rates of clinical pregnancy and live birth compared to using micronized progesterone gel alone (18). A randomized trial of 80 women with male factor infertility undergoing IVF and FET compared oral dydrogesterone to vaginal progesterone for LPS and found no difference in their effectiveness (19). Simon et al., in a retrospective study involving 171 fresh single blastocyst transfers, investigated the potential benefits of adding IM progesterone to oral dydrogesterone during FET. The study found that both groups were comparable in terms of implantation rate, early pregnancy rate, miscarriage rate, and ongoing pregnancy rate (20). In 2 large-scale clinical trials involving over 2000 participants, each found no significant difference in pregnancy rates at the 12 ${\;}^{\text{th}}$ wk or live birth rates between dydrogesterone and MVP (vaginal capsule and vaginal gel) in fresh ART cycles (21, 22).

To our knowledge, fewer studies have directly compared IM and oral progesterone for luteal support in FET (23, 24). In a retrospective study of women undergoing single FET in a tertiary center IVF unit, Bachar compared pregnancy outcomes in 2 groups receiving MVP supplemented with either dydrogesterone (10 mg 3 times daily) or IM progesterone (100 mg every 3 days) (23). While the group receiving MVP with oral dydrogesterone had lower rates of both biochemical and clinical pregnancy compared to the group receiving MVP with IM progesterone, this difference was not statistically significant. Miscarriage rates were similar between both groups. These findings are consistent with our results.

## 5. Conclusion

Our study found no statistically significant difference in clinical outcomes between women who received oral progesterone and those who received IM progesterone for LPS during FET cycles. Given the ease of use of oral dydrogesterone, it has the potential to replace the need for daily IM progesterone injections. However, the retrospective design of the study may limit our ability to establish a clear cause-and-effect relationship.

##  Data Availability

Data supporting the findings of this study are available upon reasonable request from the corresponding author.

##  Author Contributions

M. Eftekhar: Study design. M. Eftekhar, S. Arabian, S. Dashti, N. Homayoon, E. Nikfarjam: Conducted data collection and analysis. Every author contributed to the literature review, helped in drafting the manuscript, gave their approval to the finished version of the manuscript, and assumed accountability for the data's integrity.

##  Conflict of Interest

The authors declare that there is no conflict of interest.
